# Radial Head Dislocation with Ipsilateral Proximal Shaft of Radius Fracture: A Case Report

**DOI:** 10.31729/jnma.4991

**Published:** 2020-06

**Authors:** Ayush Adhikari, Subi Acharya, Ravi Bhandari

**Affiliations:** 1 Department of Orthopedics, Nepalese Army Institute of Health Sciences, Shree Birendra Hospital, Chhauni, Kathmandu, Nepal

**Keywords:** *fracture*, *ipsilateral*, *proximal shaft*, *radial head*

## Abstract

Radial head dislocations are uncommon in adults. They are commonly seen in children and are generally associated with proximal ulna fracture. Radial head dislocation with associated proximal radial shaft fracture is rarer than isolated radial head dislocation in adults. Due to the rarity of this complex injury, in the absence of keen observation and meticulous attention, the correct diagnosis might be missed leading to unsatisfactory management and related complications. Here, a similar case of radial head dislocation with associated proximal radial shaft fracture has been presented.

## INTRODUCTION

Radial head dislocations generally occur in children in association with fracture of the proximal shaft of the ulna. This is known as Monteggia fracture-dislocation.^1^

Radial head dislocations are not common in adults and only a few cases have been reported. Radial head dislocation in association with fracture of the proximal shaft of the radius is even rarer. Due to the rare occurrence of the injury, there are no definitive guidelines to manage the case. We report a case of radial head dislocation in association with the proximal shaft of radius fracture.

## CASE REPORT

A right-handed 34-year-old female presented with complaints of severe pain and swelling in her right elbow joint and forearm following a fall injury earlier that day. She fell from a height and landed on her elbow.

At the time of presentation, physical examination revealed swelling over the right elbow extending to the proximal part of the forearm and localized tenderness along proximal radial shaft. The range of motion along the elbow joint was restricted.

**Figure 1:**
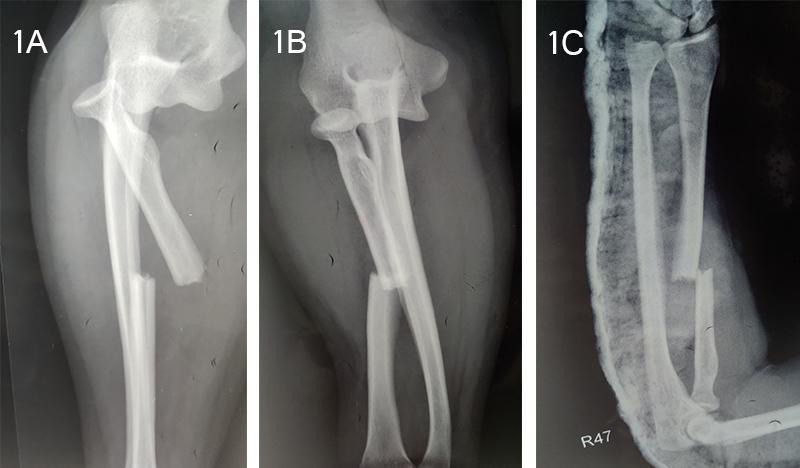
A, B, C. Plain radiographs showing radial head dislocation with ipsilateral radial shaft fracture (A) Lateral view (B) AP view (C) Immediate postoperative Xray following closed reduction of the radial head dislocation.

Plain radiographs of the region revealed posterior dislocation of the radial head associated with transverse fracture located at the junction between the upper and middle thirds of the radial shaft and the distal end was displaced posteromedially. However, the distal radio-ulnar joint remained undisturbed. The ulnar bone had no fracture (Figure 1).

**Figure 2:**
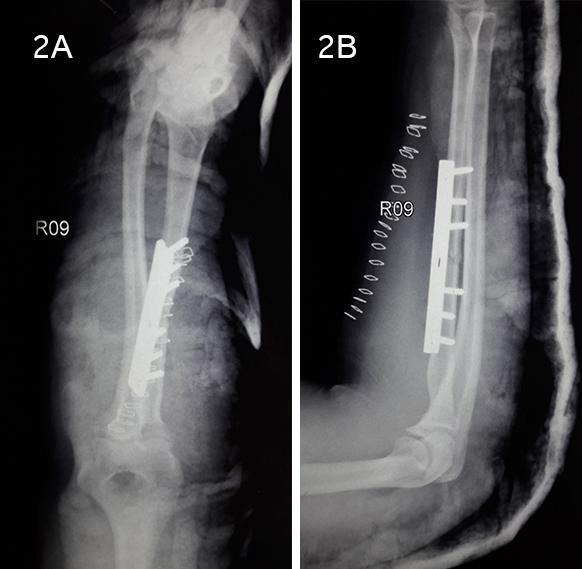
A, B. Immediate postoperative plain radiographs after radial head reduction and fixation of shaft fracture (A) Anteroposterior view (B) Lateral view.

After clinical examination and radiological findings, a diagnosis of “Radial head dislocation with the ipsilateral proximal shaft of radius fracture” was made. The patient was explained about her condition and management approach. Informed consent was taken and closed reduction under IV anaesthesia was performed. Stability was checked and the above elbow slab was applied for 3 days. Closed reduction allowed a stable elbow but the fracture ends were still displaced. So, after 3 days, the fracture ends were fixed through “Volar Henry Approach” (Figure 2). Postoperatively the limb was immobilized and the patient was kept under analgesics.

The limb was immobilized in an above-elbow cast for 6 weeks with the elbow flexed at a right angle and the forearm in supination. Range of motion exercises was started after 6 weeks.

**Figure 3:**
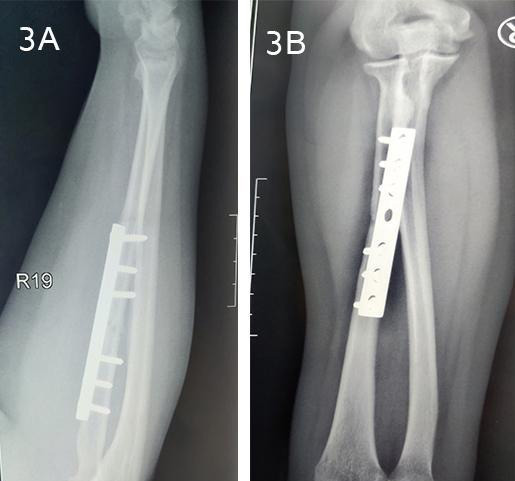
A, B. Plain radiographs at 6 months follow-up (A) Lateral view (B) Anteroposterior view.

On follow-up after 6 months, the patient was pain-free and had regained full range of movement. The radial head remained stable, and the full range of elbow movements and forearm rotation were regained (Figure 3).

## DISCUSSION

Radial head dislocations are a rarity in adults. They generally occur in association with proximal ulna fracture in children.^1^ Isolated radial head dislocation has been reported but only a few cases of radial head dislocation with associated proximal radius fracture have been reported in adults.

An anteroinferior radial head dislocation with radial shaft fracture was seen in a 25 years old male as reported by Mehara et al.^2^ A 39-year-old male also had a similar injury.^3^ There is also a reported case of missed radial head dislocation in a similar case of complex fracture-dislocation in a young adult male.^4^ Our patient was a 34-year-old lady who had posterior dislocation of the radial head. Similar complex injury has also been seen in a 4-year-old child.^5^

Annular ligament, quadrate ligament, and proximal half of the interosseous membrane are important factors in maintaining the stability of radial head which are damaged during the injury.^6^ Various mechanisms have been described as the cause. It is generally caused by high-energy trauma like a motor vehicle accident or significant falls where force is directed onto an outstretched, pronated arm.^7^ Cherif et al. reported similar injury after hyper pronation of forearm with hyperflexion of the elbow.^3^ Other mechanisms of injury include hyperextension of the elbow with the forearm in the mid prone position.^8^ In our case, the patient sustained the injury as she fell from a height and landed on the elbow with the forearm in the pronated state.

As the injury complex is rare, there are no established guidelines to manage the case. The dislocation was reduced by manipulation after open reduction of the radial shaft fracture.^3^ In a missed case of radial head dislocation in which proximal radial shaft fracture was already fixed with a dynamic compression plate, closed reduction of radial head was done successfully by Singh et al.^4^ In a reported case of four and half-year-old child by Hayami et al. open reduction of the radial shaft was done first via Henry’s approach after which radial head was reduced.^5^ In our case, a closed reduction of radial head was done first and then radial shaft fracture was fixed with DCPs after 3 days. Thus, appropriate concentric reduction of the radial head followed by fixation of fracture ends with appropriate plates and screws can be done in such injury.

Due to the rare occurrence of this kind of complex injury, good clinical examination, and meticulous attention to details while examining radiographs can thus be emphasized so as not to miss correct diagnosis and management plan. Timely diagnosis and appropriate management will prevent the complications that may arise if some injury is missed.
